# Unique Rhizobial Communities Dominated by *Bradyrhizobium liaoningense* and *Bradyrhizobium ottawaense* were Found in Vegetable Soybean Nodules in Osaka Prefecture, Japan

**DOI:** 10.1264/jsme2.ME22081

**Published:** 2023-04-11

**Authors:** Chikage Minakata, Sawa Wasai-Hara, Satori Fujioka, Shuji Sano, Atsushi Matsumura

**Affiliations:** 1 Graduate School of Life and Environmental Sciences, Osaka Prefecture University, Sakai, Osaka 599–8531, Japan; 2 National Agriculture and Food Research Organization (NARO), Tsukuba, Ibaraki 305–8604, Japan; 3 School of Life and Environmental Sciences, Osaka Prefecture University, Sakai, Osaka 599–8531, Japan; 4 Research Institute of Environment, Agriculture and Fisheries, Osaka Prefecture, Habikino 583–0862, Japan

**Keywords:** vegetable soybean, ITS region, indigenous rhizobia, *Bradyrhizobium ottawaense*, *Bradyrhizobium liaoningense*

## Abstract

Vegetable soybean (*Glycine max* [L.]) is mainly consumed in Asian countries, but has recently attracted attention worldwide due to its high nutritional value. We aimed to identify the indigenous rhizobia of vegetable soybean in Yao City, Osaka Prefecture, Japan, and to clarify the relationships between the rhizobial community and soil environmental factors. Soil samples were collected from 12 vegetable soybean cultivation fields under two different conditions (six greenhouses and six open fields) in Yao City with different varieties of vegetable soybean. A total of 217 isolates were obtained from the nodules and clustered into nine operational taxonomic units (OTUs) with 97% homology based on the 16S-23S rRNA internal transcribed spacer (ITS) region. A phylogenetic ana­lysis showed that OTUs were closely related to *Bradyrhizobium liaoningense*, *B. ottawaense*, *B. elkanii*, and other *Bradyrhizobium* species and were dominant in this order. *B. liaoningense* was widely found in sampled sites and accounted for 50.7% of all isolates, while *B. ottawaense* was mostly limited to open fields. This rhizobial community differed from Japanese soybean rhizobia, in which *B. diazoefficiens*, *B. japonicum*, and *B. elkanii* were dominant. These results imply the characteristic differences among host plants or regional specialties. A non-metric multidimensional scaling (NMDS) ana­lysis revealed the significant impact of soil pH and the contents of Ca, Mg, Mn, total nitrogen (TN), and total carbon (TC) on the distribution of rhizobia. *B. liaoningense* was detected in soils with a neutral pH, and high TN and low Mn contents increased its abundance. The present study provides novel insights into Japanese rhizobia and potentially novel resources for sustainable agriculture.

Nitrogen fixation activity is important for supplying the nitrogen demand of soybean (*Glycine max* [L.] Merr.). *Bradyrhizobium*, a Gram-negative bacterium belonging to the *Alphaproteobacteria* family, is a major symbiont of soybean. The host range of this species is wide and includes important legume crops, such as cowpea (*Vigna unguiculata* L. Walp.), faba beans (*Vicia faba* L.), and peanuts (*Arachis hypogaea*) (Santos *et al.*, 2019). Nitrogen-fixing bacteria, including *Bradyrhizobium*, fix approximately 200‍ ‍kg‍ ‍N‍ ‍ha^–1^‍ ‍year^–1^ (Herridge *et al.*, 2008), and between 60 and 80% of the nitrogen required for soybean growth is derived from nitrogen fixation. The number of species in the genus *Bradyrhizobium* has increased in recent years, revealing its vast genomic diversity (Shamseldin *et al.*, 2017; Avontuur *et al.*, 2019; Ormeño-Orrillo and Martínez-Romero, 2019). In addition to N_2_ fixation, some *Bradyrhizobium* species exhibit unique characteristics related to plant growth and the global environment. *B. elkanii* has been shown to synthesize indole-3-acetic acid (IAA), a compound belonging to the auxin family, and promotes symbiosis with its host plant (Fukuhara *et al.*, 1994; Torres *et al.*, 2018). *B. elkanii* also synthesizes rhizobitoxine and induces plant chlorosis in soybean (Owens and Wright, 1965). While some species exhibit hydrogenase activity, which increases the efficiency of nitrogen fixation (Black *et al.*, 1994), *B. cosmicum* and related strains possess photosynthetic genes (Avontuur *et al.*, 2019; Wasai-Hara *et al.*, 2020b). In addition, *B diazoefficiens* possesses the nitrous oxide reductase gene *nosZ*, which mitigates the emission of N_2_O, a known greenhouse gas (Itakura *et al.*, 2013; Akiyama *et al.*, 2016).

Rhizobial diversity in soybean fields in Japan ranges among regions (Saeki *et al.*, 2006). Only *B. japonicum*, *B. diazoefficiens*, and *B. elkanii* have been reported as major indigenous rhizobacteria in soybean fields in Japan. *B. japonicum* was found to be dominant in northern Japan and *B. elkanii* in southern Japan (Suzuki *et al.*, 2008; Saeki *et al.*, 2013). In addition, *B. japonicum* was dominant in Andosol and *B. diazoefficiens* in Graysol (Shiina *et al.*, 2014). *B. elkanii*, *B. japonicum*, *B. liaoningense*, *B. yuanmingense*, and *Ensifer* have frequently been detected as soybean rhizobia in China (Man *et al.*, 2008; Li *et al.*, 2011). *B. japonicum* and *B. diazoefficiens* are the dominant species of soybean symbionts in South Africa (Naamala *et al.*, 2016). Environmental factors contributing to genotypic differences in rhizobia include soil temperature, pH, salt, moisture content, and geographic location (Suzuki *et al.*, 2008; Zhang *et al.*, 2011; Adhikari *et al.*, 2012; Saeki and Shiro, 2014; Mason *et al.*, 2017; Yang *et al.*, 2018). Differences have been reported in the compatibility of Rhizobacteria strains with their host plants as well as their growth-promoting effects. Therefore, stronger strains are generally selected as inoculants to increase legume yields. Effective rhizobacteria, such as SEMIA 587 (*B. elkanii*), SEMIA 5079 (*B. japonicum*), and SEMIA 5080 (*B. diazoefficiens*), have been used as inoculants for soybean cultivation in Brazil (Castro-Sowinski *et al.*, 2007; de Souza *et al.*, 2019).

Vegetable soybeans are immature soybeans harvested during the R6 stage (Fehr *et al.*, 1971). They are consumed in various Asian countries and are known as *edamame* in Japan and *maodou* in China. Vegetable soybeans have recently been attracting increasing attention due to their high contents of protein, fat, phospholipids, calcium, iron, zinc, vitamins A, C, K, and B, and dietary fiber (Takahashi and Ohyama, 2011; Jiang *et al.*, 2018).

Although the total cultivation area of vegetable soybeans in Osaka Prefecture is limited, average yield is higher than in other regions in Japan (MAFF, 2020). Yao City is the main production area for vegetable soybeans in Osaka Prefecture and vegetable soybeans harvested in this city are marketed as a regional brand called “Yao-Edamame”. Yield fluctuations have been reported in Yao City, and surveys conducted in 2017 and 2018 revealed vegetable soybean yields under greenhouse conditions that ranged between 2.99 and 9.68 t ha^–1^ (Matsumura *et al.*, 2022). Therefore, it is important to evaluate various aspects, including rhizobial symbiosis, contributing to stable vegetable soybean cultivation.

Many studies have been conducted on the identification and diversity of indigenous rhizobacteria in soybean fields (Santos *et al.*, 1999; Abaidoo *et al.*, 2007; Shiro *et al.*, 2013); however, limited information is currently available on rhizobial diversity in vegetable soybean fields. Therefore, the objectives of the present study were to identify the indigenous rhizobia of vegetable soybeans in multiple fields in Yao City, Osaka Prefecture, Japan, and to compare the diversity of rhizobia among these fields. Moreover, the present study investigated the relationships between the diversity of indigenous rhizobia and environmental factors, such as soil chemical characteristics and the effects of inoculations on plant growth.

## Materials and Methods

### Soil sampling and chemical ana­lysis

**Fig. 1.**
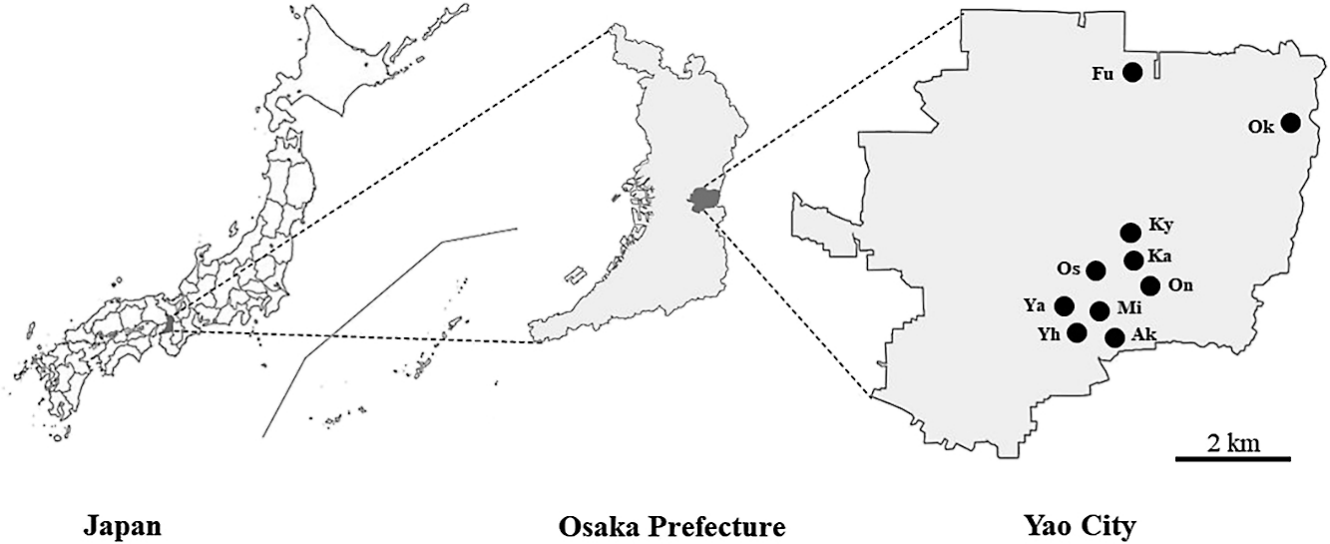
Map of Yao City in Osaka Prefecture indicating soil sampling sites. Soil samples were collected from 12 sites (6 greenhouse fields and 6 open fields), marked with black dots.

Yao City is located in east-central part of Osaka Prefecture (34°62′68″N, 135°60′9″E), Japan, and has a thriving production of vegetable soybeans. Twelve vegetable cultivation sites, including six greenhouse fields (Yaogikita [Ya], Osakabe [Os1], Fukuei-cho [Fu], Yaogihigashi-kita [Yh], Okubo [Ok1], and Kyoukouji [Ky])‍ ‍and six open fields (Kashimura-cho [Ka], Osakabe [Os2], Onjikita-machi [On], Akegawahigashi-kita [Ak], Okubo [Ok2], and Miyakoduka [Mi]) in Yao City were selected for the present study (Fig. 1). Information on soybean cultivation at each site is shown in Table 1.

Approximately 1‍ ‍kg of soil was collected from each site in 2017. During the harvesting of vegetable soybeans, soil samples were collected near the plant base between 1 to 10‍ ‍cm from the soil surface. Soil samples collected from five points in each field were composited into one sample. Soil subsamples were thoroughly mixed and air-dried before being subjected to chemical ana­lyses.

pH (H_2_O) and electrical conductivity (EC) were measured sequentially with a glass electrode (Horiba F-53 and Horiba DS-51, respectively) using a 1:5 (w/v) suspension of soil and distilled water. Nitrate-N (NO_3_^–^-N) concentrations were measured using the CATALDO method (Cataldo *et al.*, 1975). Available phosphate (P) was extracted using the Truog–P method, and its concentration was measured using the molybdenum blue colorimetric method. To assess the concentrations of soil exchangeable cations, we extracted potassium (K), calcium (Ca), magnesium (Mg), and manganese (Mn) using ammonium acetate (pH 7.0) and measured their contents using a polarized Zeeman atomic absorption spectrophotometer (ZA3000; Hitachi). Total carbon (TC) and total nitrogen (TN) contents were measured using the dry combustion method (CN coder MT-700, Yanagimoto). Similarly, soil samples were also collected before vegetable soybean cultivation, and soil chemistry data were obtained as described above. Some soil data before cultivation were previously reported by Matsumura *et al.* (2022).

### Isolation of indigenous rhizobia

To isolate indigenous rhizobia, the vegetable soybean varieties cv. Taisetsu-midori and cv. Ezo-midori were used as host plants for greenhouse and open-field soils, respectively. Taisetsu-midori and Ezo-midori are common varieties in Yao City in each type of cultivation field. Plants were grown without fertilizer in pots filled with soil (470–500 g) collected from each site. Five seeds were surface sterilized with 0.5% sodium hypochlorite for two min, rinsed five times with sterilized water, and then sown in each pot. The pots were covered with plastic bags to prevent contamination. After emergence, seedlings were thinned to accommodate two plants pot^–1^. Plants were grown in a greenhouse without temperature control, and sterile water was supplied to the soil as needed. When plants reached the V4–V5 developmental stage, 20 nodules plant^–1^ were randomly collected from the roots. Nodules were surface sterilized by immersion in 70% ethanol and 0.5% sodium hypochlorite solution for 1‍ ‍min. Nodules were carefully crushed in sterile water using toothpicks. The liquid obtained was then applied to yeast mannitol agar (YMA) (agar, 15 g; mannitol, 10 g; yeast extract, 0.4 g; KH_2_PO_4_, 0.5 g; MgSO_4_·7H_2_O, 0.2 g; NaCl, 0.1 g; distilled water, 1 L; pH 6.8) medium and incubated at 28°C for 7 days. Bacterial colonies were repeatedly streaked onto YMA medium to obtain pure cultures.

### Identification of indigenous rhizobial isolates

Colonies were selected with sterilized loops and placed directly into PCR tubes as DNA templates. An approximately 900-bp internal transcribed spacer (ITS) region between the 16S and 23S rRNA genes from the colonies obtained was amplified using colony PCR with primers from the 3′-end of 16S rRNA (FGPS1490: 5′-TGCGGCTGGATCACCTCCTT-3′) and the 5′-end of 23S rDNA (FGP132′: 5′-CCGGGTTTCCCCATTCGG-3′) (Laguerre *et al.*, 1994). PCR amplification was performed using Gflex (Takara Bio). The reaction mixture was incubated at 94°C for 5‍ ‍min, followed by 38 cycles at 94°C for 30‍ ‍s, 55°C for 30‍ ‍s, and 72°C for 2‍ ‍min, and then at 72°C for 10‍ ‍min.

PCR-amplified products were purified using the Fast Gene TM gel/PCR extraction kit (NIPPON Genetics). Sanger sequencing was performed using primers (FGPS1490 and FGP132) from Eurofins Genomics.

After checking the sequence electropherogram, samples containing contamination and/or sequencing errors were removed. Clustering was conducted using CD-Hit-est (Huang *et al.*, 2010) with a threshold of 97%, and representative sequences were used for a phylogenetic ana­lysis. A phylogenetic tree was constructed by MEGA X software (Kumar *et al.*, 2018, version 10.2.6, https://www.megasoftware.net/) using the neighbor-joining method with conserved regions of the representative sequences. A clustering ana­lysis with a threshold of 100% was also performed to establish whether isolates were identical, and isolates from the same plant host with 100% homology in the ITS sequence were defined as close isolates.

ITS sequences were deposited in the NCBI GenBank database under accession numbers ON500436 to ON500498 and OP038215. Draft genomes for representative strains of OTU1-4 were identified by shotgun sequencing using DNBSEQ-G400 (MGI Tech) at Bioengineering Lab. Genome sequences were deposited in the DDBJ database under BioSample accession numbers SAMD00570452 to SAMD00570455. Based on this genome information, average nucleotide identity (ANI) values were calculated (FastANI, Jain *et al.*, 2018) and a detailed phylogenic ana­lysis was conducted using the 31 AMPHORA genes identified (Wu and Eisen, 2008). Detailed methods for the genome ana­lysis are described in the supplemental materials.

### Inoculation effects of rhizobial isolates on vegetable soybean growth

An inoculation test using an isolate selected from each OTU was conducted to evaluate its effects on the early growth of vegetable soybeans. Plant boxes were filled with 250‍ ‍mL sterilized vermiculite and 150‍ ‍mL B&D liquid medium (Broughton and Dilworth, 1971). Soil pH and EC after the application of B&D medium were 7.0 and 0.1 dS m^–1^, respectively. This experiment consisted of ten treatments for the nine test isolates (YHC11 [OTU1], YFG21 [OTU2], YHB32 [OTU3], YHC43 [OTU4], YHC52 [OTU5], YHF61 [OTU6], YFL71 [OTU7], YFH81 [OTU8], and YHA91 [OTU9], see Table S1) and a non-inoculation (NI) treatment with five replicates. Test isolates were incubated in yeast mannitol broth (YMB) (mannitol, 10 g; yeast extract, 0.4 g; KH_2_PO_4_, 0.5 g; MgSO_4_·7H_2_O, 0.2 g; NaCl, 0.1 g; distilled water, 1 L; pH 6.8) at 28°C with reciprocal shaking at 120‍ ‍rpm for 7 days. The rhizobial solution was washed, and optical density at 660‍ ‍nm (OD_660_) was adjusted to approximately 0.3. The seeds of vegetable soybeans (cv. Ezo-midori) were surface-sterilized by immersion in 0.5% sodium hypochlorite (NaClO) for 1‍ ‍min and then washed three times with sterilized distilled water. At sowing, seeds were inoculated with a diluted solution of each test isolate (1‍ ‍mL seed^–1^). Five inoculated or non-inoculated seeds were sown in each plant box using a sterilized spoon and then thinned to one plant at the stage of primary leaf development. After thinning, the soil surface was covered with plastic wrap to prevent contamination. Plants were grown under the following controlled environmental conditions: temperature, 28°C; photoperiod, 16‍ ‍h light/8‍ ‍h dark; light intensity, 220‍ ‍μmol m^–2^s^–1^, and sterilized distilled water supplied as needed. After 22 days, plants approximately at the V4 stage were harvested and the dry weights of the shoot, root, and nodules were measured after drying at 70°C for 48 h.

The total number of nodules (NN) plant^–1^ was counted before nodule drying.

### Data ana­lysis

We used the Shannon–Wiener index (*H’*) and Simpson index (*D*) to estimate the diversity of bradyrhizobial communities. *H’* shows diversity in consideration of species richness in the community, and *D* indicates species dominance in the community. *H’* and *D* were calculated using the following equations (Mpepereki and Wollum, 1991; Ricotta and Szeidl, 2006).

*H’*=–Σ (*Pi* log *Pi*)

*D*=1–Σ*Pi*^2

*Pi* is the fraction of individuals belonging to the i^th^ species.

Additionally, relationships between these diversity indexes, soil characteristics, the occupancy of rhizobial operational taxonomic units (OTUs), and the yield of vegetable soybeans were evaluated using Pearson’s correlation. A non-metric multidimensional scaling (NMDS) ana­lysis was performed with the ‘metaMDS’ function to elucidate whether environmental variables affected rhizobial distributions. The ‘envfit’ function in ‘vegan’ was used to examine the relationships between OTU compositions and the soil and chemistry. The NMDS ana­lysis was conducted by R software using ‘vegan’ package 2.6-2 (Oksanen *et al.*, 2022). In the inoculation examination, results were subjected to Tukey’s test and Dunnett’s test comparing the NI treatment at 5% significance after an ana­lysis of variance (ANOVA). Statistical ana­lyses were performed using IBM SPSS Statistics version 26 (IBM).

## Results

### Genetic diversity and distribution of indigenous rhizobia

**Fig. 2.**
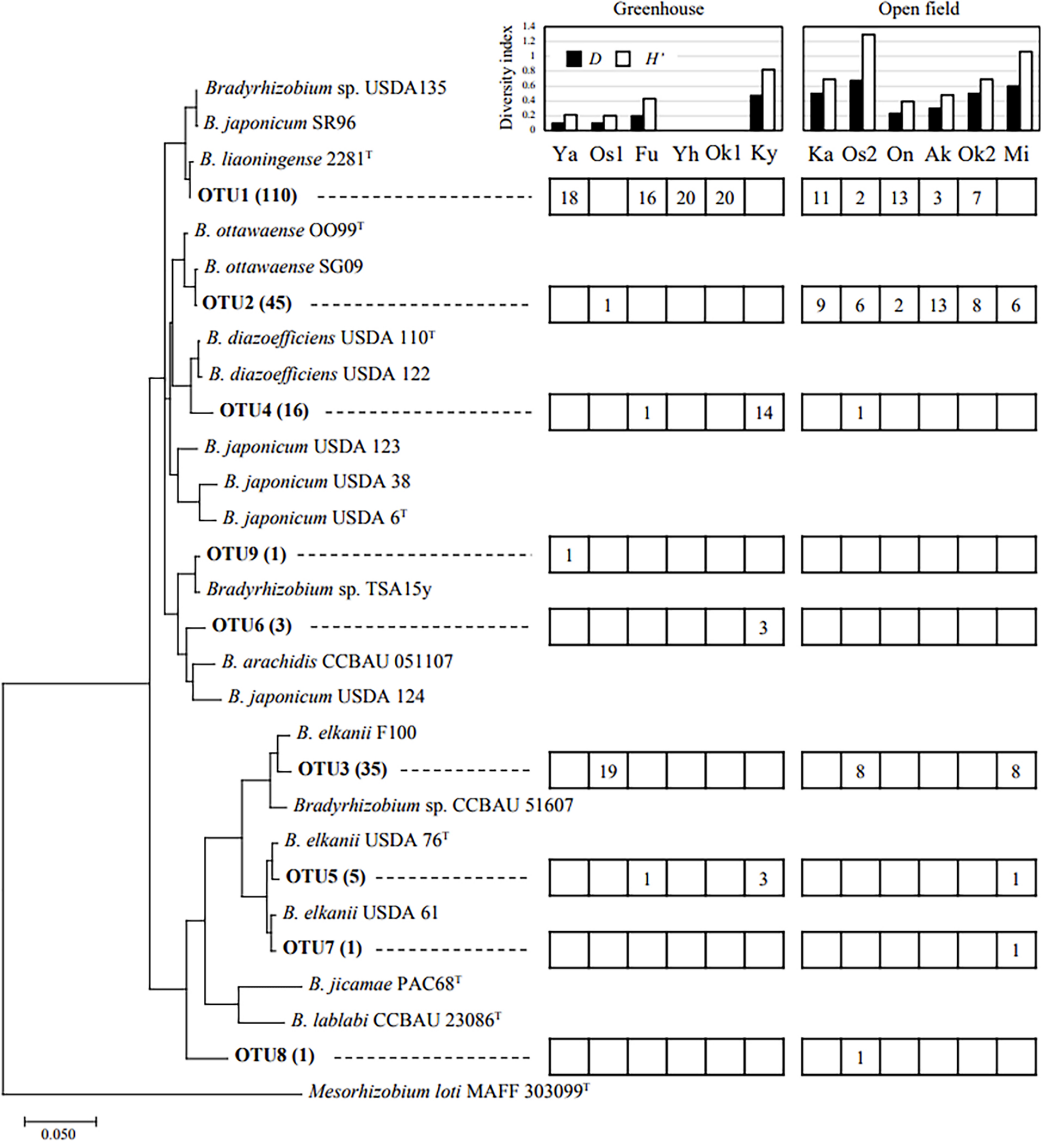
Phylogenic tree of isolates based on ITS sequences. OTU was clustered with a threshold of 97%. The numbers in parentheses show the total number of isolates included in each OTU. The right boxes show the number of isolates in each field.

A total of 217 pure rhizobial isolates were obtained from 12 soybean cultivation fields in Yao City. The results of the BLAST ana­lysis using 16S–23S ITS sequencing showed that all isolates belonged to the genus *Bradyrhizobium*. Based on a cluster ana­lysis of the ITS region, isolates were classified into nine OTUs with 97% homology (Fig. 2) and 31 genotypes with 100% homology (Table S1, 6 genotypes in OTU1, 5 in OTU2, 4 in OTU3, 8 in OTU4, 2 in OTU 5, 3 in OTU6, and 1 in OTU7-9).

Genome Average Nucleotide Identity (ANI) values calculated between representative strains of OTU1 (YHC11), OTU2 (YFG21), OTU3 (YHB32), and OTU4 (YHC43) and closely related strains showed that each isolate belonged to *B. liaoningense*, *B. ottawaense*, *B. elkanii*, and *B. diazoefficiens*, respectively (Table S2). A detailed phylogenic ana­lysis based on AMPHORA genes supported this classification (Fig. S1).

The most commonly detected species in Yao City was OTU1 (50.7%), closely related to *B. liaoningense*, followed by OTU2 (20.7%), closely related to *B. ottawaense* (Fig. 2). OTU1 was distributed regardless of whether the sites were in greenhouses or open fields. On the other hand, OTU2 was abundant in all open fields, but was limited in greenhouse fields. OTU3 (16.1%), closely related to *B. elkanii* F100, was detected in three fields. Site Os1, with a high dominance of OTU3, was the only greenhouse field in which Green 80 was cultivated instead of Taisetsu-Midori (Table 1). OTU4 (7.4%), closely related to *B. diazoefficiens* USDA 110^T^ and USDA 122, was distributed in three sites. The percentage of OTU4 at site Ky was high. OTU5 (2.3%), closely related to *B. elkanii* USDA 76^T^, was distributed at three sites, but was less dominant at all sites. OTU6 (1.4%), closely related to *B. arachidis*, was less dominant, being detected in only three isolates. OTU7 (0.5%), closely related to *B. elkanii* USDA 61, OTU8 (0.46%), without closely related isolates, and OTU9 (0.46%), closely related to *Bradyrhizobium sp.* TSA15y, were distributed in only one of the sites. OTU8 comprised a single isolate, YFH81, and the BLAST search showed the highest homology to the *B. japonicum* strain SR96 (Fig. 2), but at a low value (91.4%), and its exact classification remains unclear.

Using the number of each bradyrhizobial OTU, we calculated two diversity indexes, Simpson (*D*) and Shannon–Wiener (*H’*) in each site (Fig. 2). The highest *D* was found at site Os2 (0.67), followed by site Mi (0.60). The lowest *D *value (0) was found at sites Yh and Ok1, where only OTU1 was isolated. Similar results were obtained for the diversity index *H’*, with the highest *H’* at site Os2 (1.29), followed by site Mi (1.06), and the lowest *H’* (0) at sites Yh and Ok1. The correlation ana­lysis revealed no correlations between each diversity index and soil chemistry (data not shown).

The marketable pod yield of vegetable soybeans ranged between 5.13 and 8.68 t ha^–1^ among the greenhouse fields surveyed (Matsumura *et al.*, 2022) and between 3.31 and 12.07 t ha^–1^ among the open fields (Table 1), revealing a large yield variation among the fields. In the present study, no correlations were observed between vegetable soybean yield and rhizobial OTU occupancy or diversity indexes.

### Relationship between the distribution of each OTU and soil chemistry

Soil chemistry data at the harvesting of vegetable soybeans are shown in Table 2. Overall, soil pH was higher in the open fields, while EC and the content of Mg were higher in greenhouse fields. Regarding the other factors tested, large differences were noted among fields regardless of whether they were open or greenhouse fields.

**Fig. 3.**
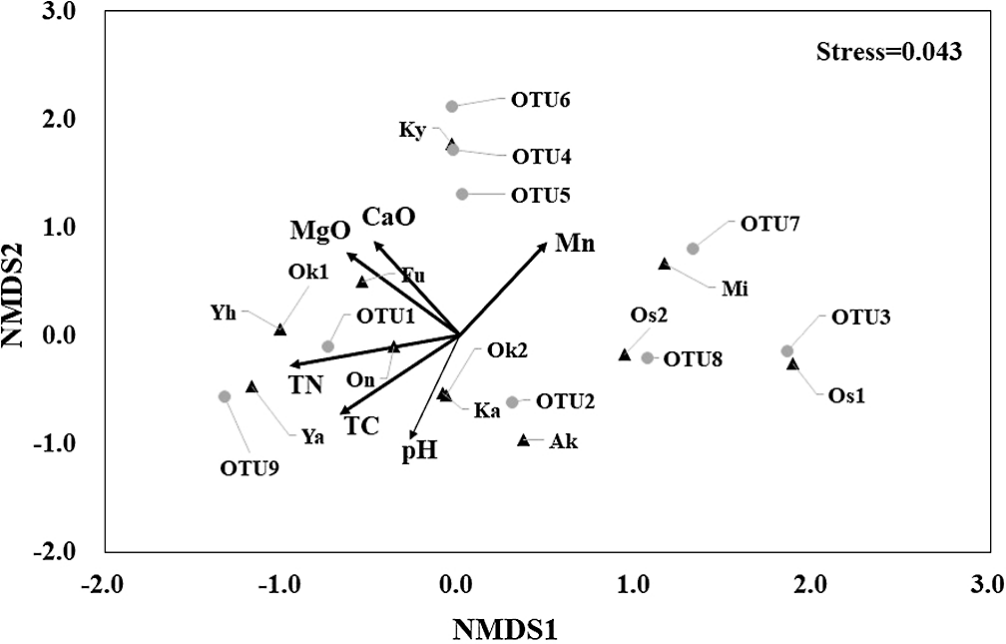
Non-metric multidimensional scaling (NMDS) ordination of rhizobial community structures (circles) and soil sampling sites (triangles). Arrows represent vectors of significant environmental variables explaining the ordination (*P*<0.05).

The NMDS ordination of rhizobial communities showed a low stress level (0.043), indicating a good representation of OTU compositions (Fig. 3). The NMDS ordination did not show a clear separation of sites according to the cultivation method or field type. There was a trend for sites to cluster together according to OTU abundance, for example, a high OTU1 group (sites Ya, Fu, Yh, Ok1, and On) and high OTU2 group (sites Ka, Ak, and Ok2). Three sites, Os2, Mi, and Os1, in which OTU3 closely related to *B. elkanii* F100 was detected, positioned right side, while site Ky, in which OTU4 closely related to *B. diazoefficiens* was identified, was clearly separated to upper side.

Ten soil chemical factors that fit as vectors in the NMDS ana­lysis showed that pH and the contents of Ca, Mg, Mn, TC, and TN significantly contributed to the distribution of OTUs (Fig. 2 and Table 3). Important factors related to the distribution of OTUs were soil pH (*P*=0.003, *R*^2^=0.765) and the content of Ca (*P*=0.004, *R*^2^=0.760) (Table 3). The main species OTU1 was detected in soils with a neutral pH and high TN and TC contents. The next main species OTU2 was found under various soil conditions. In contrast, OTU3 and OTU4 were identified in slightly acidic soils with a higher Mn content. OTU1, OTU4, OTU5, and OTU6 were detected in soils with high Ca and Mg contents. The minor species, OTU7 and OTU8 were positioned near OTU3 and OTU9 near OTU1.

The NMDS ordination of rhizobial OTUs and soil chemistry before vegetable soybean cultivation showed that pH and the contents of Ca, Mg, Mn, and P significantly contributed to the distribution of OTUs (Fig. S1). These results were similar to those of the NMDS ana­lysis using soil collected at harvesting (Fig. 3).

### Inoculation examination

**Fig. 4.**
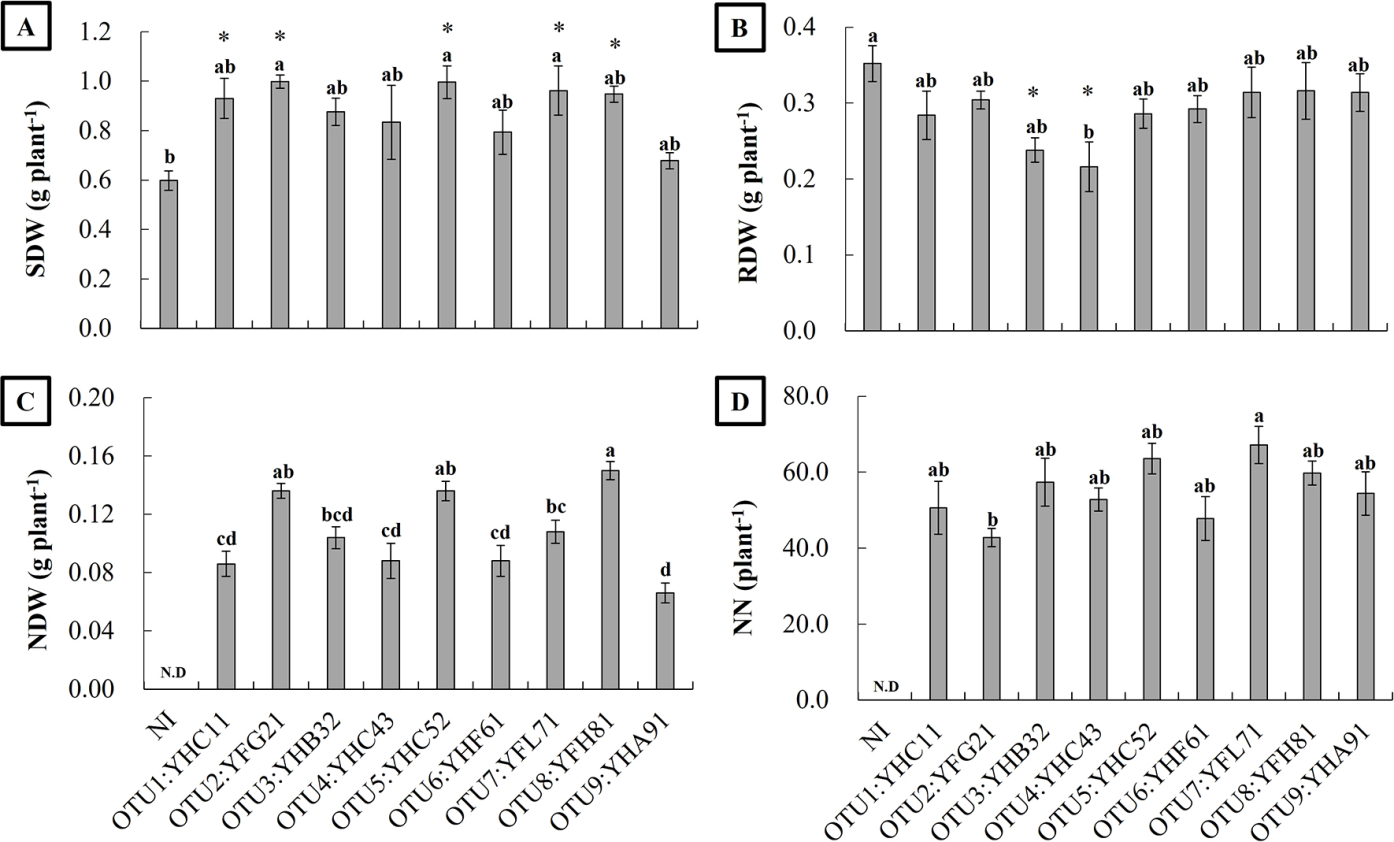
Effects of each test strain inoculation on shoot dry weight (SDW) (A), root dry weight (RDW) (B), nodule dry weight (NDW) (C), and the number of nodules (NN) (D) of vegetable soybeans under controlled conditions. Values are averages (*n*=5)±SE (error bars). Averages followed by different letters are significantly different at *P*<0.05 between the inoculation treatment analyzed using Tukey’s test, after an ana­lysis of variance (ANOVA), and averages followed by * are significantly different from the non-inoculation (NI) treatment at *P*<0.05 analyzed using Dunnett’s test.

Vegetable soybeans (cv: Ezo-midori) were inoculated with nine representative isolates obtained from the 97% homology classification, and the following phenotypes were evaluated at the V4 stage. Shoot dry weights (SDWs) significantly differed among the treatments (Fig. 4A). Growth-promoting effects on shoot growth varied among the isolates: the growth of vegetable soybeans inoculated with YFG21 (OTU2), THC52 (OTU5), and YFL71 (OTU7) was significantly better than those with the NI treatment. On the other hand, no significant differences were observed between the non-inoculated control and inoculations with YHC11 (OTU1), YHB32 (OTU3), YHC43 (OTU4), YHF61 (OTU6), YFH81 (OTU8), and YHA91 (OTU9) (Fig. 4A). Root dry weights (RDWs) were slightly lower with inoculations than with the NI treatment. The inoculation with YHC43 (OTU4) significantly inhibited root growth (Fig. 4B). Nodule dry weights (NDWs) varied among the isolates, particularly those inoculated with YFH81 (OTU8), which were significantly higher than those inoculated with the other test isolates, except for YFG21 (OTU2) and YHC52 (OTU5) (Fig. 4C). NN did not significantly differ among the test isolates; however, YFL71 (OTU7) formed the most nodules and YFG21 (OTU2) the fewest (Fig. 4D). Some isolates, such as YFG21 (OTU2), had low NN, but high NDWs, and the size of the nodules formed by the inoculated isolates varied. In comparisons of OTU1 and OTU3, the dominant isolates in Yao City, the nodule size of YFG21 (OTU2) was larger than that of YHC11 (OTU1) or YHB32 (OTU3).

## Discussion

### Relationship between rhizobial communities and soil chemical properties

In the present study, indigenous rhizobacteria, such as *B. liaoningense*, *B. ottawaense*, *B. elkanii*, and *B. diazoefficiens*, were isolated from vegetable soybean fields in Yao City. *B. liaoningense* and *B. ottawaense* were highly distributed in the surveyed fields. *B. liaoningense* was detected in both greenhouse and open fields, whereas *B. ottawaense* was mostly restricted to open fields (Fig. 2).

*B. liaoningense*, which had the highest dominance in vegetable soybean cultivation fields, was originally isolated from soybean fields in Liaoning and other provinces in China (Xu *et al.*, 1995; Zhang *et al.*, 2011). It was also isolated from alkaline-saline soils and was reportedly tolerant of high pH (Appunu *et al.*, 2008; Li *et al.*, 2011). In the present study, *B. liaoningense* was detected in fields with neutral soil (pH 6.3–7.3), and was absent from those with acidic soil (pH 5.3–6.0), suggesting that it is not suited to thrive in acidic soils. In the NMDS ana­lysis (Fig. 3) fitting significant soil chemical properties (Table 2), the abundance of *B. liaoningense* was slightly higher in fields with high TN soils, particularly in greenhouse fields at sites Ya, Fu, Yh, and Ok1. At *B. liaoningense*-dominant sites, soil EC was higher than the average. In contrast, a high Mn content appeared to negatively affect the abundance of *B. liaoningense*. Based on previous studies and the present results, *B. liaoningense* may be adapted to more fertile soils under neutral to high pH conditions during vegetable soybean cultivation.

*B. ottawaense*, the second most dominant species (Fig. 2), was originally isolated from soybean fields in Ottawa, Canada (Yu *et al.*, 2014). It has been also isolated from soybean and peanut cultivation fields in China, and the distribution of *B. ottawaense* has been reported to positively correlate with available nitrogen and the TN content in soil (Yan *et al.*, 2017; Shao *et al.*, 2020). In the present study, *B. ottawaense* was detected under various soil nutrient conditions: soil pH (6.0–7.3), soil nitrate (1.0–121.8‍ ‍mg kg^–1^), available phosphate (219.9–4397.0‍ ‍mg kg^–1^), K_2_O (42.3–199.9‍ ‍mg kg^–1^), Mn (2.5–49.6‍ ‍mg kg^–1^), and TN (0.04–0.12‍ ‍mg g^–1^), suggesting that it may widely adapt to different soil environments.

*B. elkanii*, reportedly the main indigenous *Bradyrhizobium* species in Japan, was isolated from three sites (Os1, Os2, and Mi) (Fig. 2). These three sites had low soil EC, TN contents, and nitrate-N concentrations, which suggested the preference of *B. elkanii* for soils with low fertility. Previous studies reported that *B. elkanii* was dominant in acidic soils (Zhang *et al.*, 2011; Suzuki *et al.*, 2014). Since the surveyed fields in the present study generally had neutral soil, soil characteristics other than pH may have affected the distribution of *B. elkanii*. *B. elkanii* was previously found to be dominant in fields with low soil fertility (Herrmann *et al.*, 2014), which is consistent with the present results.

OTU4, closely related to *B. diazoefficiens*, was mostly limited to the Ky site. In Japan, *B. diazoefficiens* USDA110 appeared to be dominant in reductive soils with low permeability, such as rice paddy conversion fields (Shiina *et al.*, 2014; Saeki and Shiro, 2014; Saeki *et al.*, 2017). However, site Ky is a recently developed upland with soil dressing, not an upland rice paddy conversion field. As a major feature, site Ky had the lowest soil pH (5.3) among the fields surveyed. Tu *et al.* (2021) isolated acid-tolerant rhizobial bacteria closely related to *B. diazoefficiens* USDA 110^T^ from soybean nodules in Taiwan, and identified them as potential inoculants to induce the nodulation of soybeans in strongly acidic soils. These findings are consistent with the present results; therefore, OTU4 closely related to *B. diazoefficiens* may be dominant in site Ky.

The closely related isolate of *B. elkanii*, OTU5 was also detected in fields with low fertility, similar to OTU3. Furthermore, site Ky, from which OTU5 was isolated, had a significantly higher Ca content in its soil than in the other study plots, suggesting that *B. elkanii* prefers Ca-rich soils. Previous studies (Zhang *et al.*, 2022) suggested that *B. arachidis* was isolated from acidic soils and was more likely to be distributed in soils with low fertility, which is consistent with the soil chemistry characteristics of the Ky site, from which OTU6 closely related to *B. arachidis* was isolated. Since only one strain was detected in OTU7, OTU8, and OTU9, this cannot be definitively discussed in the present study.

### Inoculation effects of indigenous *Bradyrhizobium* in Yao City

In the present study, no correlations were observed between the farmer’s yield and the occupancy rate of each rhizobial OTU. However, since different farmers in different fields cultivate vegetable soybeans using different methods, difficulties are associated with obtaining accurate data. We‍ ‍herein investigated the effects of *B. liaoningense* (YHC11[OTU1]), *B. ottawaense* (YFG21 [OTU2]), *B. elkanii* (YHB32 [OTU3], YHC52 [OTU5], and YFL71 [OTU7]), *B. diazoefficiens* (YHC43 [OTU4]), *B. arachidis* (YHF61 [OTU6]), *Bradyrhizobium sp.* (YHA91 [OTU9]), and YFH81 (OTU8) on the early growth of vegetable soybean cv. Ezo-midori. The results obtained showed that shoot growth was significantly greater with some rhizobia (YFG21 [OTU2], YHC52 [OTU5], and YFL71 [OTU7]) than with the non-inoculated treatment, but was limited in early growth evaluations (Fig. 4). Therefore, further studies are needed to examine the effects of each test isolate on the yield of vegetable soybeans under the same field conditions.

### Unique rhizobial communities in vegetable soybeans in Yao City

The rhizobial communities identified in the present study are unique from those described in previous studies conducted on soybean fields in Japan (Saeki *et al.*, 2006; Saeki *et al.*, 2013) because *B. liaoningense* and *B. ottawaense* were dominant (Fig. 2). *B. ottawaense* was only reported to have been isolated from the roots of sorghum in Japan (Wasai-Hara *et al.*, 2020a), and no studies showed that *B. liaoningense* was dominant in soybean nodules. Therefore, to the best of our knowledge, the present study is the first to isolate *B. liaoningense* and *B. ottawaense* from soybean roots in Japan. Interestingly, *B. ottawaense* was detected almost exclusively in open field soils, while *B. liaoningense* was identified in both greenhouse and open field soils. Although geographic location has been reported to affect the distribution of *Bradyrhizobium* (Shiro *et al.*, 2013), these differences were less effective in the present study because the surveyed fields were located in a very small area (Fig. 1 and Table 1). Temperature sensitivity may affect the dominance of these rhizobia because vegetable soybeans were grown between February and May in greenhouses and between April and August in open fields in Yao City. Zhang *et al.* (2003) and Suzuki *et al.* (2014) reported that the nodulation and competitiveness of *Bradyrhizobium* were affected by soil temperature, and tolerance of low temperatures varied among rhizobial species. However, an incubation test conducted under low temperatures showed no evidence of *B. liaoningense*, which was dominant in greenhouse fields, being tolerant of low temperatures, suggesting that the low temperature tolerance of each indigenous rhizobium did not affect the distribution of *Bradyrhizobium* in Yao City (Fig. S2). Although crop rotation may affect indigenous rhizobia, burdock was cultivated as a previous crop in most fields (Table 1) indicating little effect on differences in the dominance of *B. liaoningense* and *B. ottawaense*.

Other differences between greenhouse and open field cultivation is the vegetable soybean cultivar, with ‘Taisetsu-Midori’ mainly being used for greenhouse cultivation and ‘Ezo-Midori’ for open field cultivation. The effector proteins of rhizobia and the *Rj* and *NNL1* genes in host plants have been shown to affect compatibility (Yang *et al.*, 2010; Sugawara *et al.*, 2018; Zhang *et al.*, 2021). Further studies with a gene ana­lysis of host-controlled nodulation are needed to clarify the mechanisms underlying compatibility between vegetable soybeans and *B. liaoningense* and *B. ottawaense*. Furthermore, rhizobial invasion from external sources may be possible because farmers often use mountain soil as soil dressing in Yao City. Although it was unclear whether soil dressing was performed in the surveyed fields, it may have contributed to the difference in dominance between *B. liaoningense* and *B. ottawaense*.

### Potential agricultural use of major indigenous *Bradyrhizobium* in Yao City

Previous studies reported that *Ensifer melitoi*, *Rhizobium leguminosarum*, *and B. diazoefficiens* possess *nosZ*-coding N_2_O reductase, and *B. diazoefficiens* may mitigate soil N_2_O emissions during the growth and post-growth periods of soybean crops on a field scale (Itakura *et al.*, 2013; Torres *et al.*, 2014; Akiyama *et al.*, 2016; Catherine *et al.*, 2022). *B. ottawaense* was also recently shown to possess *nosZ* (Mania *et al.*, 2019; Wasai-Hara *et al.*, 2020a). Sustainable agricultural technology with a low environmental impact is highly expected in the near future. The results of the genome ana­lysis confirmed that YHC11 (OTU1) and YFG21 (OTU2), which are closely related to *B. liaoningense* and *B. ottawaense*, also possess *nosZ*. By focusing on vegetable soybean fields, the present study provides novel insights into the Japanese rhizobial flora and new materials for agriculture with less of an environmental impact.

## Citation

Minakata, C., Wasai-Hara, S., Fujioka, S., Sano, S., and Matsumura, A. (2023) Unique Rhizobial Communities Dominated by *Bradyrhizobium liaoningense* and *Bradyrhizobium ottawaense* were Found in Vegetable Soybean Nodules in Osaka Prefecture, Japan. *Microbes Environ ***38**: ME22081.

https://doi.org/10.1264/jsme2.ME22081

## Supplementary Material

Supplementary Material

## Figures and Tables

**Table 1.** Information on vegetable soybean cultivation at each soil sampling site in Yao City, 2017

	Site				Vegetable soybean cultivar	Amount of fertilization (kg ha^–1^)			marketable pods yield (t ha^–1^)	previous crop
			Latitude (°N)	Longitude (°E)		N	P_2_O_5_	K_2_O		
**Greenhouse**	Yaogi-kita	Ya	34.365	135.365	Taisetsu-midori	168	196	140	8.15	Burdock
	Osakabe	Os1	34.365	135.371	Green 80	67	22	14	8.68	Burdock
	Fukuei	Fu	34.643	135.627	Taisetsu-midori	126	42	27	5.59	Japanese mustard spinach
	Yaogi-higashi	Yh	34.363	135.374	Taisetsu-midori	22	30	13	5.13	Burdock
	Okubo	Ok1	34.374	135.392	Taisetsu-midori	16	22	10	8.09	Burdock
	Kyoukouji	Ky	34.377	135.376	Taisetsu-midori	73	100	43	7.41	Burdock
**Open field**	Kashimura	Ka	34.365	135.373	Ezo-midori	168	200	140	7.28	Burdock
	Osakabe	Os2	34.365	135.371	Ezo-midori	38	13	8	7.37	Spinach
	Onjikita	On	34.364	135.376	Ezo-midori	13	4	3	8.87	Spinach
	Akegawa-higashi	Ak	34.364	135.365	Ezo-midori	43	14	9	12.07	Burdock
	Okubo	Ok2	34.374	135.392	Ezo-midori	18	24	10	3.31	Burdock
	Miyakoduka	Mi	34.362	135.372	Ezo-midori	68	92	40	7.63	Green onion

**Table 2.** Soil characteristics of 12 sample sites after vegetable soybean harvesting

	Site	pH (H_2_O)	EC		NO_3_-N	P_2_O_5_	K_2_O	CaO	MgO	Mn		TC	TN
			(dS m^–1^)		(mg kg^–1^)							(mg g^–1^)	
Greenhouse	Ya	6.6	0.4		121.8	2,968.7	405.2	2,265.2	576.8	4.4		1.66	0.18
	Os1	6.0	0.1		17.9	219.9	181.1	896.0	205.1	21.8		0.81	0.05
	Fu	6.3	0.8		90.2	1,544.6	52.3	2,976.4	366.7	2.4		1.08	0.14
	Yh	6.7	0.7		28.7	1,496.5	98.0	3,427.2	369.8	11.7		1.08	0.12
	Ok1	6.6	0.7		84.1	2,977.0	124.0	3,014.2	423.1	6.8		1.63	0.18
	Ky	5.3	0.1		41.8	130.5	54.1	5,471.2	665.5	37.7		0.73	0.07
Open field	Ka	7.3	0.1		68.7	3,317.8	199.9	1,638.0	384.3	2.5		1.21	0.12
	Os2	6.6	0.1		10.2	313.3	172.6	1,289.4	252.9	11.6		1.04	0.04
	On	6.9	0.1		43.3	1,636.2	49.4	2,564.8	155.7	5.1		0.85	0.08
	AK	7.0	0.1		121.8	4,397.0	87.9	2,459.8	158.9	3.0		1.13	0.11
	Ok2	6.5	0.1		26.0	2,367.3	42.3	2,198.0	157.1	7.7		1.40	0.10
	Mi	6.0	0.2		1.0	2,880.3	181.0	1,940.4	146.9	49.6		1.12	0.10
**Average**		6.5	0.3		54.6	2,020.8	137.3	2,511.7	321.9	13.7		1.14	0.11

**Table 3.** Linear correlation (*R*^2^) of soil environmental variables in the NMDS ana­lysis. The significance of relationships was calculated using 999 permutations. Bold values indicate significant soil environmental variables (*P*<0.05).

**Variable**	**R^2^**	***P *** **value**
**pH**	**0.765**	**0.003**
EC	0.422	0.074
NO_3_-N	0.429	0.080
P_2_O_5_	0.386	0.101
K_2_O	0.083	0.694
**CaO**	**0.760**	**0.004**
**MgO**	**0.529**	**0.031**
**Mn**	**0.741**	**0.007**
**TC**	0.486	0.048
**TN**	**0.622**	**0.008**

## References

[B1] Abaidoo, R.C., Keyser, H.H., Singleton, P.W., Dashiell, K.E., and Sanginga, N. (2007) Population size, distribution, and symbiotic characteristics of indigenous *Bradyrhizobium* spp. that nodulate TGx soybean genotypes in Africa. Appl Soil Ecol 35: 57–67.

[B2] Adhikari, D., Kaneto, M., Itoh, K., Suyama, K., Pokharel, B.B., and Gaihre, Y.K. (2012) Genetic diversity of soybean-nodulating rhizobia in Nepal in relation to climate and soil properties. Plant Soil 357: 131–145.

[B3] Akiyama, H., Takada-Hoshino1, Y., Itakura, M., Shimomura, Y., Wang, Y., Yamamoto, A., et al. (2016) Mitigation of soil N_2_O emission by inoculation with a mixed culture of indigenous *Bradyrhizobium diazoefficiens*. Sci Rep 6: 32869. 27633524 10.1038/srep32869 PMC5025649

[B4] Appunu, C., N’Zoue, A., and Laguerre, G. (2008) Genetic diversity of native bradyrhizobia isolated from soybeans (*Glycine max* L.) in different agricultural-ecological-climatic regions of India. Appl Environ Microbiol 74: 5991–5996. 18676699 10.1128/AEM.01320-08 PMC2565974

[B5] Avontuur, J.R., Palmer, M., Beukes, C.W., Chan, W.Y., Coetzee, M.P.A., Blom, J., et al. (2019) Genome-informed *Bradyrhizobium* taxonomy: where to from here? Syst Appl Microbiol 42: 427–439. 31031014 10.1016/j.syapm.2019.03.006

[B6] Black, L.K., Fu, C., and Maier, R.J. (1994) Sequences and characterization of *hupU* and *hupV* genes of *Bradyrhizobium japonicum* encoding a possible nickel-sensing complex involved in hydrogenase expression. J Bacteriol 176: 7102–7106. 7961478 10.1128/jb.176.22.7102-7106.1994 PMC197088

[B63] Broughton, W.J. and Dilworth, M. (1971) Control of leghaemoglobin synthesis in snake beans. Biochem J 125: 1075-1080. 5144223 10.1042/bj1251075 PMC1178271

[B7] Castro-Sowinski, S., Herschkovitz, Y., Okon, Y., and Jurkevitch, E. (2007) Effects of inoculation with plant growth-promoting rhizobacteria on resident rhizosphere microorganisms. FEMS Microbiol Lett 276: 1–11. 17711454 10.1111/j.1574-6968.2007.00878.x

[B8] Cataldo, D.A., Haroon, M., Scharader, L.E., and Youngs, V.L. (1975) Rapid colorimetric determination of nitrate in plant tissue by nitration of salicylic acid. Commun Soil Sci Plant Anal 6: 71–80.

[B9] Catherine, H., Barbier, E., Alain, H., and Cecile, R. (2022) New insights into the use of rhizobia to mitigate soil N_2_O emissions. Agriculture 12: 271.

[B11] de Souza, G.K., Sampaio, J., Longoni, L., Ferreira, S., Alvarenga, S., and Beneduzi, A. (2019) Soybean inoculants in Brazil: an overview of quality control. Braz J Microbiol 50: 205–211. 30637629 10.1007/s42770-018-0028-z PMC6863340

[B12] Fehr, W.R., Caviness, C.E., Burmood, D.T., and Pennington, J.S. (1971) Stages of development descriptions for soybeans [*Glycine max* (L.) Merrill]. Crop Sci 11: 929–931.

[B13] Fukuhara, H., Minakawa, Y., Akao, S., and Minamisawa, K. (1994) The involvement of indole-3-acetic acid produced by *Bradyrhizobium elkanii* in nodule formation. Plant Cell Physiol 35: 1261–1265.

[B14] Herridge, D.F., Peoples, M.B., and Boddey, R.M. (2008) Global inputs of biological nitrogen fixation in agricultural systems. Plant Soil 311: 1–18.

[B15] Herrmann, L., Chotte, J.L., Thuita, M., and Lesueur, D. (2014) Effects of cropping systems, maize residues application and N fertilization on promiscuous soybean yields and diversity of native rhizobia in Central Kenya. Pedobiologia 57: 75–85.

[B16] Huang, Y., Niu, B., Gao, Y., Fu, L., and Li, W. (2010) CD-HIT Suite: a web server for clustering and comparing biological sequences. Bioinformatics 26: 680–682. 20053844 10.1093/bioinformatics/btq003 PMC2828112

[B17] Itakura, M., Uchida, Y., Akiyama, H., Hoshino, Y.T., Shimomura, Y., Morimoto, S., et al. (2013) Mitigation of nitrous oxide emissions from soils by *Bradyrhizobium japonicum* inoculation. Nat Clim Change 3: 208–212.

[B18] Jain, C., Rodriguez-R, L.M., Phillippy, A.M., Konstantinidis, K.T., and Aluru, S. (2018) High throughput ANI ana­lysis of 90K prokaryotic genomes reveals clear species boundaries. Nat Commun 9: 5114. 30504855 10.1038/s41467-018-07641-9 PMC6269478

[B19] Jiang, G.L., Rutto, L.K., Ren, S., Bowen, R.A., Berry, H., and Epps, K. (2018) Genetic ana­lysis of edamame seed composition and trait relationships in soybean lines. Euphytica 214: 158.

[B20] Kumar, S., Stecher, G., Li, M., Knyaz, C., and Tamura, K. (2018) MEGA X: Molecular evolutionary genetics ana­lysis across computing platforms. Mol Biol Evol 35: 1547–1549. 29722887 10.1093/molbev/msy096 PMC5967553

[B21] Laguerre, G., Allard, M.R., Revoy, F., and Amarger, N. (1994) Rapid identification of rhizobia by restriction fragment length polymorphism ana­lysis of PCR-amplified 16S rRNA genes. Appl Environ Microbiol 60: 56–63. 16349165 10.1128/aem.60.1.56-63.1994 PMC201269

[B22] Li, Q.Q., Wang, E.T., Zhang, Y.Z., Zhang, Y.M., Tian, C.F., Sui, X.H., et al. (2011) Diversity and biogeography of rhizobia isolated from root nodules of *Glycine max* grown in Hebei Province, China. Microb Ecol 61: 917–931. 21340735 10.1007/s00248-011-9820-0

[B23] Man, C.X., Wang, H., Chen, W.F., Sui, X.H., Wang, E.T., and Chen, W.X. (2008) Diverse rhizobia associated with soybean grown in the subtropical and tropical regions of China. Plant Soil 310: 77–87.

[B24] Mania, D., Woliy, K., Degefu, T., and Frostegård, Å. (2019) A common mechanism for efficient N_2_O reduction in diverse isolates of nodule-forming bradyrhizobia. Environ Microbiol 22: 17–31. 31271499 10.1111/1462-2920.14731

[B25] Mason, M.L.T., Matsuura, S., and Domingo, A.L. (2017) Genetic diversity of indigenous soybean-nodulating *Bradyrhizobium elkanii* from southern Japan and Nueva Ecija, Philippines. Plant Soil 417: 349–362.

[B26] Matsumura, A., Sano, S., Ueda, Y., Yamasaki, M., and Tokumoto, H. (2022) Variabilities in agronomic traits and their relationship with soil properties of vegetable soybean cultivated under greenhouse conditions in the Nakakawachi region, Osaka, Japan. Hortic J 91: 356–365.

[B27] Ministry of Agriculture, Forestry and Fisheries (MAFF). (2020) Annual report on food, agriculture and rural areas in Japan. URL https://www.e-stat.go.jp/stat-search/files?page=1&layout=datalist&toukei=00500215&tstat=000001013427&cycle=7&year=20200&month=0&tclass1=000001032286&tclass2=000001032933&tclass3=000001161149&tclass4val=0

[B28] Mpepereki, S., and Wollum, A.G. (1991) Diversity of indigenous *Bradyrhizobium japonicum* in North Carolina soils. Biol Fertil Soils 11: 121–127.

[B29] Naamala, J., Jaiswal, S.K., and Dakora, F.D. (2016) Microsymbiont diversity and phylogeny of native bradyrhizobia associated with soybean (*Glycine max* L. Merr.) nodulation in South African soils. Syst Appl Microbiol 39: 336–344. 27324571 10.1016/j.syapm.2016.05.009 PMC4958686

[B30] Oksanen, J., Simpson, G.L., Blanchet, F.G., Kindt, R., Legendre, P., Minchin, P.R., *et al.* (2022) Ordination methods, diversity ana­lysis and other functions for community and vegetation ecologists. R package version 2.6-2. URL https://cran.r-project.org/web/packages/vegan/index.html

[B31] Ormeño-Orrillo, E., and Martínez-Romero, E. (2019) A genomotaxonomy view of the bradyrhizobium genus. Front Microbiol 10: 1–13. 31263459 10.3389/fmicb.2019.01334 PMC6585233

[B32] Owens, L.D., and Wright, D.A. (1965) Production of the soybean-chlorosis toxin by *Rhizobium japonicum* in pure culture. Plant Physiol 40: 931–933. 16656177 10.1104/pp.40.5.931 PMC550408

[B33] Ricotta, C., and Szeidl, L. (2006) Towards a unifying approach to diversity measures: bridging the gap between the Shannon entropy and Rao’s quadratic index. Theor Popul Biol 70: 237–243. 16904144 10.1016/j.tpb.2006.06.003

[B34] Saeki, Y., Aimi, N., Tsukamoto, S., Yamakawa, T., Nagatomo, Y., and Akao, S. (2006) Diversity and geographical distribution of indigenous soybean-nodulating bradyrhizobia in Japan. Soil Sci Plant Nutr 52: 418–426.

[B35] Saeki, Y., Shiro, S., Tajima, T., Yamamoto, A., Sameshima-Saito, R., Sato, T., et al. (2013) Mathematical ecology ana­lysis of geographical distribution of soybean-Nodulating Bradyrhizobia in Japan. Microbes Environ 28: 470–478. 24240318 10.1264/jsme2.ME13079 PMC4070701

[B36] Saeki, Y., and Shiro, S. (2014) Comparison of soybean-nodulating Bradyrhizobia community structures along north latitude between Japan and USA. In *Advances in Biology and Ecology of Nitrogen Fixation*. Ohyama, T. (ed.) IntechOpen, pp. 195–224. URL https://doi.org/10.5772/56990

[B37] Saeki, Y., Nakamura, M., Mason, M.L.T., Yano, T., Shiro, S., Sameshima-Saito, R., et al. (2017) Effect of flooding and the *nosZ* gene in bradyrhizobia on bradyrhizobial community structure in the soil. Microbes Environ 32: 154–163. 28592720 10.1264/jsme2.ME16132 PMC5478539

[B38] Santos, M.A., Vargas, M.A.T., and Hungria, M. (1999) Characterization of soybean Bradyrhizobium strains adapted to the Brazilian savannas. FEMS Microbiol Ecol 30: 261–272. 10525182 10.1111/j.1574-6941.1999.tb00654.x

[B39] Santos, M.S., Nogueira, M.A., and Hungria, M. (2019) Microbial inoculants: reviewing the past, discussing the present and previewing an outstanding future for the use of beneficial bacteria in agriculture. AMB Express 9: 205. 31865554 10.1186/s13568-019-0932-0 PMC6925611

[B40] Shamseldin, A., Abdelkhalek, A., and Sadowsky, M.J. (2017) Recent changes to the classification of symbiotic, nitrogen-fixing, legume-associating bacteria: a review. Symbiosis 71: 91–109.

[B41] Shao, S., Chen, M., Liu, W., Hu, X., and Li, Y. (2020) Long-term monoculture reduces the symbiotic rhizobial biodiversity of peanut. Syst Appl Microbiol 43: 126101. 32847777 10.1016/j.syapm.2020.126101

[B42] Shiina, Y., Itakura, M., Choi, H., Saeki, Y., Hayatsu, M., and Minamisawa, K. (2014) Relationship between soil type and N_2_O reductase genotype (*nosZ*) of indigenous soybean bradyrhizobia: *NosZ*-minus populations are dominant in andosols. Microbes Environ 29: 420–426. 25476067 10.1264/jsme2.ME14130 PMC4262367

[B43] Shiro, S., Matsuura, S., Saiki, R., Sigua, G.C., Yamamoto, A., Umehara, Y., et al. (2013) Genetic diversity and geographic distribution of indigenous soybean-nodulating bradyrhizobia in the United States. Appl Environ Microbiol 79: 3610–3618. 23563944 10.1128/AEM.00236-13 PMC3675916

[B44] Sugawara, M., Takahashi, S., Umehara, Y., Iwano, H., Tsurumaru, H., Odake, H., et al. (2018) Variation in bradyrhizobial NopP effector determines symbiotic incompatibility with *Rj2*-soybeans via effector-triggered immunity. Nat Commun 9: 3139. 30087346 10.1038/s41467-018-05663-x PMC6081438

[B45] Suzuki, K., Oguro, H., Yamakawa, T., Yamamoto, A., Akao, S., and Saeki, Y. (2008) Diversity and distribution of indigenous soybean-nodulating rhizobia in the Okinawa islands, Japan. Soil Sci Plant Nutr 54: 237–246.

[B46] Suzuki, Y., Adhikari, D., Itoh, K., and Suyama, K. (2014) Effects of temperature on competition and relative dominance of *Bradyrhizobium japonicum* and *Bradyrhizobium elkanii* in the process of soybean nodulation. Plant Soil 374: 915–924.

[B47] Takahashi, Y., and Ohyama, T. (2011) Soybeans: cultivation, uses and nutrition. In *Production and Consumption of Green Vegetable Soybeans “Edamame”*. Maxwell, J.E. (ed.) Hauppauge, NY: Nova Science Publishers, Inc., pp. 425–442.

[B48] Torres, D., Benavidez, I., Donadio, F., Mongiardini, E., Rosas, S., Spaepen, S., et al. (2018) New insights into auxin metabolism in, *Bradyrhizobium japonicum*. Res Microbiol 169: 313–323. 29751062 10.1016/j.resmic.2018.04.002

[B49] Torres, M.J., Rubia, M.I., de la Peña, T.C., Pueyo, J.J., Bedmar, E.J., and Delgado, M.J. (2014) Genetic basis for denitrification in *Ensifer melioti*. BMC Microbiol 14: 142. 24888981 10.1186/1471-2180-14-142 PMC4064527

[B50] Tu, T., Lin, S., and Shen, F. (2021) Enhancing symbiotic nitrogen fixation and soybean growth through co-inoculation with *Bradyrhizobium* and *Pseudomonas* isolates. Sustainability 13: 11539.

[B51] Wasai-Hara, S., Hara, S., Morikawa, T., Sugawara, M., Takami, H., Yoneda, J., et al. (2020a) Diversity of *Bradyrhizobium* in non-leguminous sorghum plants: *B. ottawaense* isolates unique in genes for N_2_O reductase and lack of the type VI secretion system. Microbes Environ 35: ME19102. 31932539 10.1264/jsme2.ME19102 PMC7104290

[B52] Wasai-Hara, S., Minamisawa, K., Cloutier, S., and Bromfield, E.S.P. (2020b) Strains of *Bradyrhizobium cosmicum* sp. nov., isolated from contrasting habitats in Japan and Canada possess photosynthesis gene clusters with the hallmark of genomic islands. Int J Syst Evol Microbiol 70: 5063–5074. 32804606 10.1099/ijsem.0.004380 PMC7656271

[B53] Wu, M., and Eisen, J.A. (2008) A simple, fast, and accurate method of phylogenomic inference. Genome Biol 9: R151. 18851752 10.1186/gb-2008-9-10-r151 PMC2760878

[B54] Xu, L.M., Ge, C., Cui, Z., Li, J., and Fan, H. (1995) *Bradyrhizobium liaoningense* sp. nov., isolated from the root nodules of soybeans. Int J Syst Bacteriol 45: 706–711. 7547289 10.1099/00207713-45-4-706

[B55] Yan, J., Chen, W., Han, X., Wang, E., Zou, W., and Zhang, Z. (2017) Genetic diversity of indigenous soybean-nodulating rhizobia in response to locally-based long term fertilization in a Mollisol of Northeast China. World J Microbiol Biotechnol 33: 6. 27848139 10.1007/s11274-016-2170-9

[B56] Yang, S., Tang, F., Gao, M., and Zhu, H. (2010) *R gene*-controlled host specificity in the legume-rhizobia symbiosis. Proc Natl Acad Sci U S A 107: 18735–18740. 20937853 10.1073/pnas.1011957107 PMC2973005

[B57] Yang, S.H., Chen, W.H., Wang, E.T., Chen, W.F., Yan, J., Han, X.Z., et al. (2018) Rhizobial biogeography and inoculation application to soybean in four regions across China. J Appl Microbiol 125: 853–866. 29719942 10.1111/jam.13897

[B58] Yu, X., Cloutier, S., Tambong, J.T., and Bromfield, E.S.P. (2014) *Bradyrhizobium ottawaense* sp. nov., a symbiotic nitrogen fixing bacterium from root nodules of soybeans in Canada. Int J Syst Evol Microbiol 64: 3202–3207. 24969302 10.1099/ijs.0.065540-0 PMC4156109

[B59] Zhang, B., Wang, M., Sun, Y., Zhao, P., Liu, C., Qing, K., et al. (2021) *Glycine max NNL1* restricts symbiotic compatibility with widely distributed bradyrhizobia via root hair infection. Nat Plants 7: 73–86. 33452487 10.1038/s41477-020-00832-7

[B60] Zhang, H., Prithiviraj, B., Charles, T.C., Driscoll, B.T., and Smith, D.L. (2003) Low temperature tolerant *Bradyrhizobium japonicum* strains allowing improved nodulation and nitrogen fixation of soybean in a short season (cool spring) area. Eur J Agron 19: 205–213.

[B61] Zhang, J., Peng, S., Li, S., Song, J., Brunel, B., Wang, E., et al. (2022) Arachis hypogaea L. from acid soils of Nanyang (China) is frequently associated with *Bradyrhizobium guangdongense* and occasionally with *Bradyrhizobium ottawaense* or three Bradyrhizobium genospecies. Microb Ecol 84: 556–564. 34528105 10.1007/s00248-021-01852-2

[B62] Zhang, Y.M., Li, Y. Jr., Chen, W.F., Wang, E.T., Tian, C.F., Li, Q.Q., et al. (2011) Biodiversity and biogeography of rhizobia associated with soybean plants grown in the north China plain. Appl Environ Microbiol 77: 6331–6342. 21784912 10.1128/AEM.00542-11 PMC3187167

