# Supplementary material for: Unique Rhizobial Communities Dominated by Bradyrhizobium liaoningense and Bradyrhizobium ottawaense were Found in Vegetable Soybean Nodules in Osaka Prefecture, Japan
Source: Microbes Environ. 2023 Apr 11;38(2):ME22081. doi: 10.1264/jsme2.ME22081 (PMC10308233; doi:10.1264/jsme2.ME22081)
Supplement: Supplementary file 1 — Supplementary Material [file 38_22081_s1.pdf]

Supplemental data

Unique rhizobial communities dominated by *B. liaoningense* and *B. ottawaense* were found in vegetable soybean nodules in Osaka prefecture, Japan

Detailed methods

1. Draft genome sequence

The extraction of genomic DNA and shotgun sequences were conducted by Bioengineering Lab. Co., Ltd (Kanagawa, Japan). The DNA extraction and purification were performed using a MPure-12 system and a MPure Bacterial DNA Extraction Kit (MP Biomedicals, California, USA). DNA libraries were prepared with a MGIEasy FS DNA Library Prep Set and sequenced DNBSEQ-G400 (MGI Tech Co., Ltd.) Raw reads were trimmed in CLC Genomics Workbench v. 8.5.1 software (CLC Bio, Aarhus, Denmark) with the following parameters: ambiguous limit, 2; quality limit, 0.05; number of 5′ terminal nucleotides, 20; number of 3′ terminal nucleotides, 5; minimum number of nucleotides in reads, 70. The trimmed reads assembled in the CLC Genomic Workbench with the shortest contig at 200 bp. Annotation performed the DFAST webserver (https://dfast.ddbj.nig.ac.jp/) and deposited in NCBI GenBank.

2. Phylogenic analysis by AMPHORA genes

To analyze detailed phylogeny, amino acid sequences of 31 AMPHORA genes were extracted, then concatenated and aligned. Phylogenic tree was constructed in MEGA X (version 10.2.6, https://www.megasoftware.net/) with neighbor-joining methods with conserved region.

3. Comparison of two major indigenous *Bradyrhizobium* growth under low temperature conditions

Three representative isolates selected from OTU1 and OTU2 were used as the test isolates. We incubated each isolate in HM medium (Na_2HPO_4, 0.125 g; Na_2SO_4, 0.25 g; NH_4Cl, 0.32 g; $MgSO_4 \cdot 7H_2O$, 0.18 g; $FeCl_3$, 0.004 g; $CaCl_2 \cdot 2H_2O$, 0.013 g; HEPES, 1.3 g; MES, 1.1g; Yeast Extract, 0.25 g; L-Arabinose, 1.0 g; distilled water, 1 liter; pH 6.8) (Cole and Elkan, 1973) for 7 days at 18 or 28℃ with shaking at 140 rpm, and measured optical density at 660 nm almost every day.

Table S1. Indigenous *Bradyrhizobium* detected in soil collected from Yao City.

	Isolate	Isolated site[a]		Number of isolates	Genotype[b]	Accession number[c]
OTU1: Closely related to *B. liaoningense*	YFG11	Field	Ka	1	1-1	ON500440
	YFI11	Field	On	3	1-1	ON500449
	YFK11	Field	Ok2	7	1-1	ON500462
	YHA11	House	Ya	6	1-1	ON500474
	YHC11†	House	Fu	15	1-1	ON500493
	YHD11	House	Yh	20	1-1	ON500497
	YHE11	House	Ok1	20	1-1	OP038125
	YFG12	Field	Ka	5	1-2	ON500437
	YFI12	Field	On	2	1-2	ON500454
	YHA12	House	Ya	8	1-2	ON500475
	YHC12	House	Fu	1	1-2	ON500494
	YFH13	Field	Os2	1	1-3	ON500447
	YFI13	Field	On	5	1-3	ON500450
	YFJ13	Field	Ak	1	1-3	ON500461
	YHA13	House	Ya	1	1-3	ON500476
	YFG14	Field	Ka	4	1-4	ON500438
	YFH14	Field	Os2	1	1-4	ON500446
	YFI14	Field	On	1	1-4	ON500451
	YFJ14	Field	Ak	1	1-4	ON500459
	YFG15	Field	Ka	1	1-5	ON500441
	YFI15	Field	On	2	1-5	ON500453
	YHA15	House	Ya	3	1-5	ON500473
	YFJ16	Field	Ak	1	1-6	ON500460
	Total number of isolates			110		
OTU2: Closely related to *B. ottawaense*	YFG21	Field	Ka	7	2-1	ON500436
	YFH21†	Field	Os2	4	2-1	ON500444
	YFI21	Field	On	1	2-1	ON500455
	YFJ21	Field	Ak	6	2-1	ON500456
	YFK21	Field	Ok2	4	2-1	ON500464
	YFL21	Field	Mi	1	2-1	ON500472
	YFG22	Field	Ka	2	2-2	ON500439
	YFH22	Field	Os2	2	2-2	ON500442
	YFI22	Field	On	1	2-2	ON500452
	YFJ22	Field	Ak	6	2-2	ON500457
	YFK22	Field	Ok2	3	2-2	ON500463
	YFL22	Field	Mi	4	2-2	ON500466
	YHB22	House	Os1	1	2-2	ON500491
	YFJ23	Field	Ak	1	2-3	ON500458
	YFK24	Field	Ok2	1	2-4	ON500465
	YFL25	Field	Mi	1	2-5	ON500471
	Total number of isolates			45		
OTU3: Closely related to *B. elkanii*	YFH31	Field	OS2	8	3-1	ON500443
	YFL31	Field	Mi	7	3-1	ON500467
	YHB31	House	Os1	12	3-1	ON500490
	YHB32†	House	Os1	6	3-2	ON500489
	YFL33	Field	Mi	1	3-3	ON500470
	YHB34	House	Os1	1	3-4	ON500492
	Total number of isolates			35		
OTU4: Closely related to *B. diazoefficiens*	YFH41	Field	Os2	1	4-1	ON500445
	YHF41	House	Ky	4	4-1	ON500479
	YHF42	House	Ky	4	4-2	ON500482
	YHF43	House	Ky	1	4-3	ON500478

	YHC43	House	Fu	1	4-3	ON500495
	YHF44†	House	Ky	1	4-4	ON500487
	YHF45	House	Ky	1	4-5	ON500480
	YHF46	House	Ky	1	4-6	ON500488
	YHF47	House	Ky	1	4-7	ON500481
	YHF48	House	Ky	1	4-8	ON500484
	Total number of isolates			16		
OTU5: Closely related to *B. elkanii*	YFL51†	Field	Mi	1	5-1	ON500468
	YHF51	Field	Ky	3	5-1	ON500477
	YHC52	Field	Fu	1	5-2	ON500496
	Total number of isolates			5		
OTU6: Closely related to *B. arachidis*	YHF61	House	Ky	1	6-1	ON500483
	YHF62	House	Ky	1	6-2	ON500486
	YHF63	House	Ky	1	6-3	ON500485
	Total number of isolates			3		
OTU7: Closely related to *B. elkanii*	YFL71†	Field	Mi	1	7-1	ON500469
	Total number of isolates			1		
OTU8: without closely related isolates	YFH81†	Field	Os2	1	8-1	ON500448
	Total number of isolates			1		
OTU9: Closely related to *Bradyrhizobium* sp. TSA15y	YHA91†	House	Ya	1	9-1	ON500498
	Total number of isolates			1		

[a] Ya, Yaogikita; Os, Osakabe; Fu, Fukuei-cho; Yh, Yaogihigashi; Ok, Okubo; Ky, Kyoukouji; Ka, Kashimura-cho; On, Onjikita-machi; Mi, Miyakoduka.

[b] Isolates with 100% ITS sequence homologies are indicated by the same numbers.

[c] Accession numbers indicate that the amplicon was present and sequenced, and data were deposited in NCBI GenBank.

† Representative sequences used in the phylogenetic tree (Fig. 2).

Table S2. ANI between test isolates and reference *Bradyrhizobium* species.

Test isolate	OTU	Expected species	*B. liaoningense* CCBAU 83689	*B. ottawaense* OO99T	*B. elkanii* USDA 76T	*B. diazoefficiens* USDA 110T	YHC11	YFG21	YHB32	YHC43
YHC11	1	*B. liaoningense*	**96.27**	90.44	83.26	90.09	-	90.45	83.47	90.14
YFG21	2	*B. ottawaense*	89.77	**99.01**	83.51	90.35	90.45	-	83.60	90.67
YHB32	3	*B. elkanii*	83.11	83.68	**99.66**	83.74	83.47	83.60	-	83.77
YHC43	4	*B. diazoefficiens*	89.17	90.49	83.59	**99.86**	90.14	90.67	83.77	-

Table S3. Soil characteristics of the 12 sampling sites before vegetable soybean cultivation.

	Site	pH (H_2O)	EC ($dS\ m^{-1}$)	NO_3-N	P_2O_5	K_2O	CaO	MgO	Mn
				($mg\ kg^{-1}$)					
Greenhouse	Ya	6.6	0.52	20.3	3097.0	297.1	2286.2	71.5	3.9
	Os1	5.5	0.04	12.4	220.8	289.2	627.2	68.7	20.8
	Fu	6.5	0.74	37.1	2512.9	40.1	3340.4	458.1	4.0
	Yh	7.3	0.21	36.0	1829.1	105.1	3194.8	305.3	3.8
	Ok1	6.7	0.24	6.8	3540.4	79.7	2423.4	321.4	10.3
	Ky	5.6	0.05	9.0	147.5	68.9	4972.8	624.2	24.9
Open field	Ka	7.2	0.08	9.0	3158.4	226.3	1440.6	403.8	3.4
	Os2	5.3	0.38	87.8	415.0	460.5	922.6	137.7	23.9
	On	6.5	0.21	11.3	1800.2	131.6	2212.0	148.3	10.2
	Ak	7.1	0.06	6.8	4133.6	68.7	1955.8	67.8	1.6
	Ok2	6.7	0.09	14.6	2976.0	58.0	1906.8	128.2	4.3
	Mi	5.7	0.10	13.5	2519.8	219.2	1174.6	97.0	34.1
	Average	6.4	0.23	22.1	2195.9	170.4	2204.8	236.0	12.1

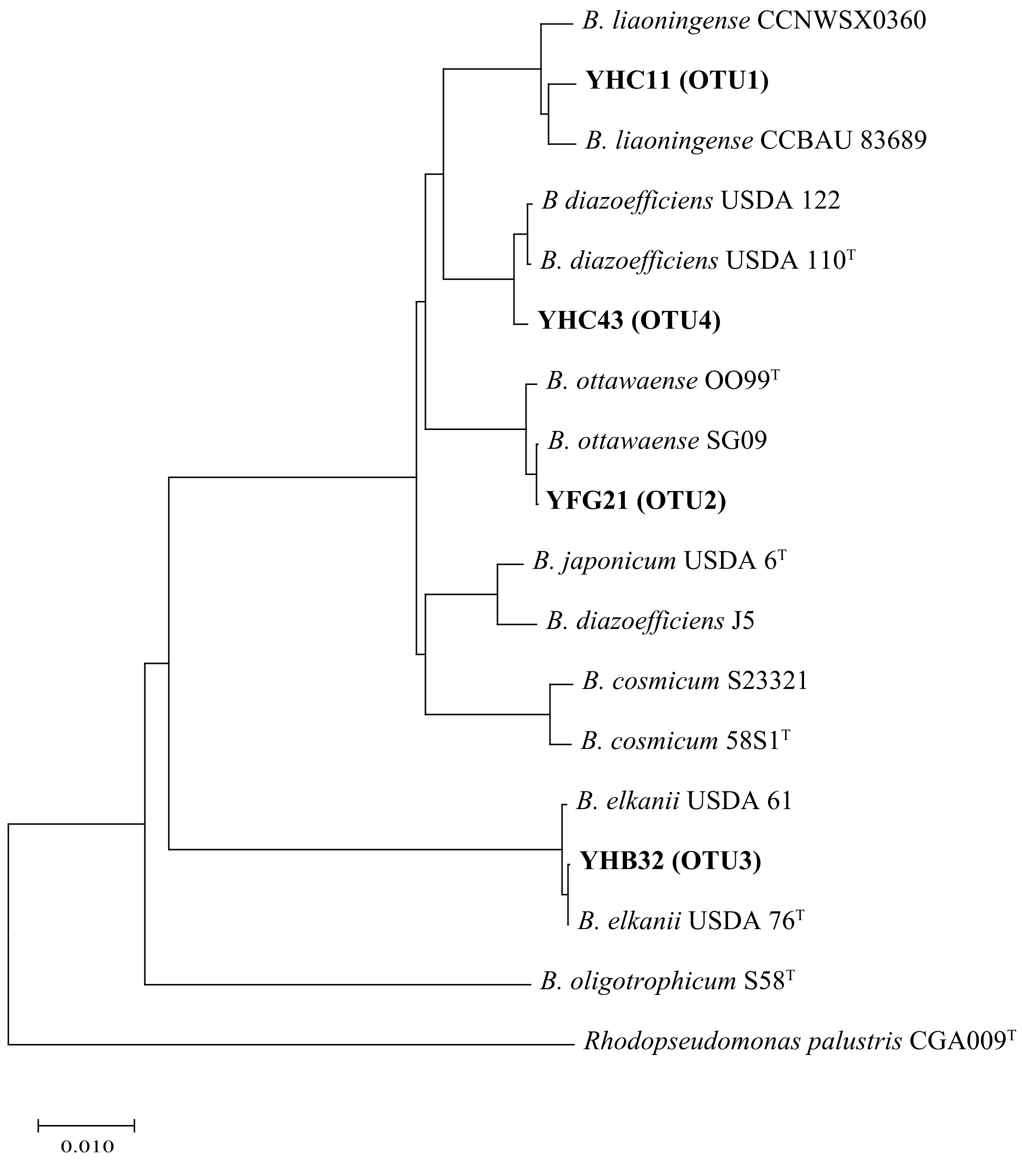

Fig. S1. Phylogenic tree of four representative strains of OTU1～4 based on 31 AMPHORA genes.

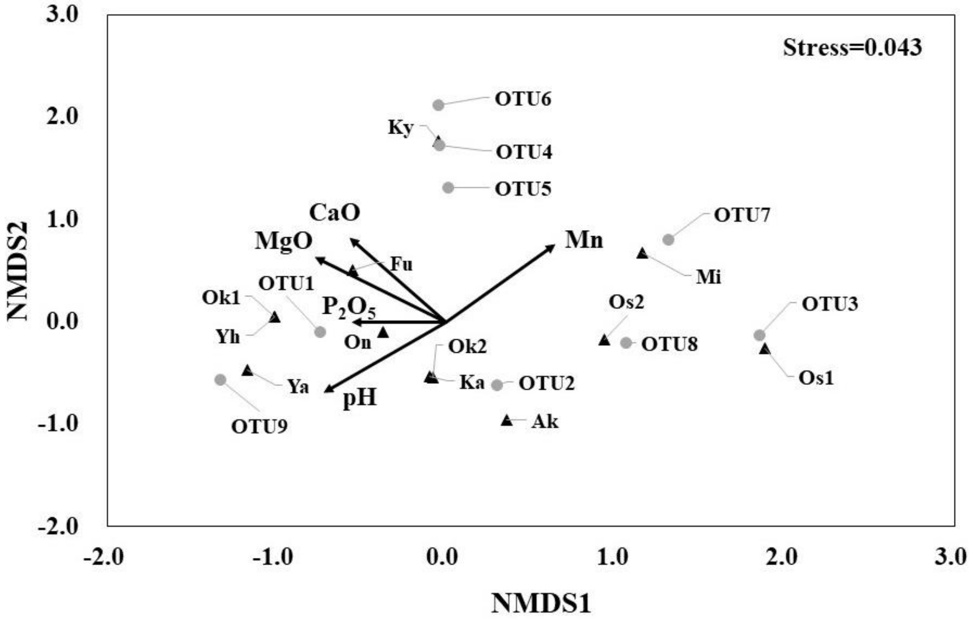

Fig. S2. Non-metric multidimensional scaling (NMDS) ordination of rhizobial community structure (circles) and soil sampling sites (triangles). Arrows represent vectors of significant pre-cultivation environmental variables explaining the ordination (p<0.05).

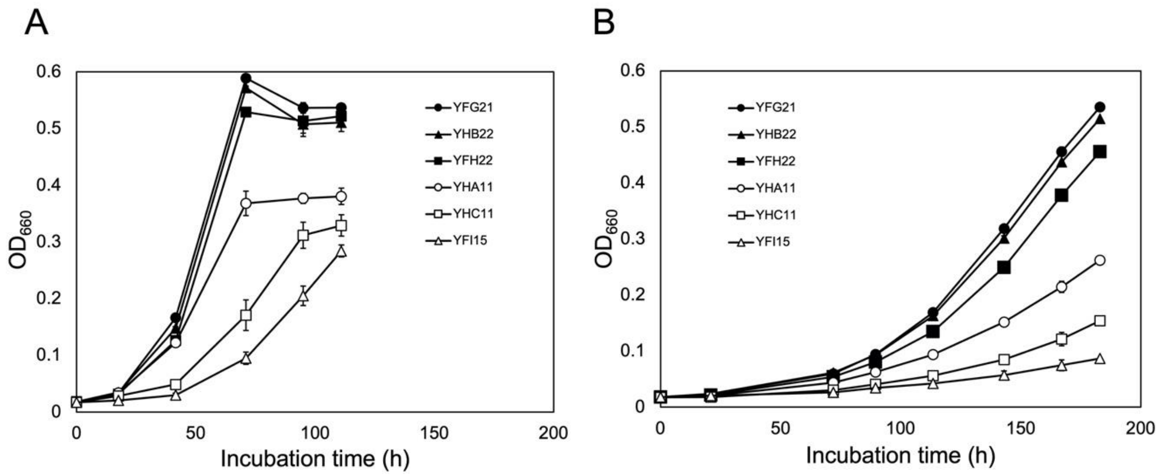

Fig. S3. Growth curves of *B. liaoningense* (open symbols, ○=YHA11, □=YHC11 (representative strains in ITS sequence), △=YFI15) and *B. ottawaense* (closed symbols, ●=YFG21 (representative strains in ITS sequence), ▲=YHB22, ■=YFH22) in HM medium at (A) 28℃ and (B) 18℃ conditions.
